# Expression status of folate receptor alpha is a predictor of survival in pancreatic ductal adenocarcinoma

**DOI:** 10.18632/oncotarget.16841

**Published:** 2017-04-05

**Authors:** Lei Cai, Theodoros Michelakos, Cristina R. Ferrone, Liyuan Zhang, Vikram Deshpande, Qi Shen, Albert DeLeo, Teppei Yamada, Gong Zhang, Soldano Ferrone, Xinhui Wang

**Affiliations:** ^1^ Division of Surgical Oncology, Department of Surgery, Massachusetts General Hospital, Harvard Medical School, Boston, MA, USA; ^2^ Department of Hepatobiliary, Southwest Hospital, Third Military Medical University, Chongqing, China; ^3^ Department of Pathology, Massachusetts General Hospital, Harvard Medical School, Boston, MA, USA; ^4^ University of Pittsburgh Cancer Institute, University of Pittsburgh, Pittsburgh, PA, USA; ^5^ Department of Orthopaedic Surgery, Massachusetts General Hospital, Harvard Medical School, Boston, MA, USA

**Keywords:** folate receptor alpha, pancreatic ductal adenocarcinoma, predictor of survival, smoking, alcohol consumption

## Abstract

Pancreatic ductal adenocarcinoma (PDAC) has one of the poorest prognosis among malignancies. Thus, the identification of markers useful in developing innovative diagnostic and therapeutic methods is an imperative need. Folate receptor alpha (FRα) has been associated with prognosis in several cancers and has served as a target of novel anti-tumor therapies. However, FRα expression in PDAC and its correlation with the clinical course of the disease has not been thoroughly investigated. In this study, we analyzed FRα expression in 140 PDAC specimens and 7 PDAC cell lines in order to define the significance of FRα expression in PDAC and its potential role as a target for immunotherapy. Immunohistochemical analysis demonstrated that FRα expression intensity was low, intermediate and high in 22(16%), 73(52%) and 45(32%) PDACs, respectively. The staining was located in both membrane and cytoplasm in most cases (123, 88%). Lower FRα expression was associated with cigarette smoking (p<0.001), alcohol consumption (p<0.001), and lymphovascular invasion (p=0.002). Additionally, lower FRα expression was associated with poor overall survival (5-year overall survival: low 13%, intermediate 31%, high 33%; p=0.006). FRα expression (HR=0.61; p=0.03) and Charlson Comorbidity Index (HR=1.16; p=0.01) emerged as independent predictors of survival. The analysis by flow cytometry of 7 PDAC cell lines (AsPC-1, Capan-2, MIA PaCa-2, PANC-1, PDAC2, PDAC3, and PDAC5) demonstrated the highest expression of FRα on the PDAC3 cell line (45%). Therefore, a higher FRα expression is predictive of a favorable prognosis in PDAC and FRα may represent a promising target for novel treatments, including immunotherapy.

## INTRODUCTION

Pancreatic Ductal Adenocarcinoma (PDAC) continues to have one of the worst outcomes of any malignancy. It is the fourth most common cause of cancer death in the United States [1, 2]. Resection is currently the only curative method, however, the 5-year overall survival rate after surgical resection is less than 5% [3]. Unfortunately, however, most patients present with advanced unresectable and/or metastatic tumors. Although major risk factors for PDAC, namely, smoking [4, 5], excessive alcohol consumption [6], meat-rich diet and diabetes [7], have been identified, diagnostic methods using specific markers to predict the occurrence of PDAC are lacking. However, the survival benefit of perioperative therapeutic modalities, such as chemotherapy and chemo-radiation therapy, has been demonstrated in large-scale randomized controlled trials. Consequently, efforts are being made to identify relevant factors and/or markers that predict a high risk of recurrence and poor prognosis, which may help to optimize perioperative therapeutic approaches for those patients with resectable PDAC [8, 9]. Clearly, it is urgent to understand the pathogenesis of PDAC to aid in the identification of markers useful in developing innovative diagnostic and therapeutic methods for this disease.

A potential marker for PDAC is Folate Receptor Alpha (FRα, also known as folate binding protein [FBP]), a glycosylphosphatidylinositol-linked protein with high affinity for folate (folic acid, or vitamin B9), which acts by an endocytosis mechanism. It belongs to one of the two classes of folate transport, the other class represented by the reduced folate carrier [10].

Three FR protein isoforms have been discovered – referred to as FRα, FRβ and FRγ– each with tissue-specific distribution and folate binding potential. At the gene level, these three FR isoforms have similar highly conserved sequences (about 70% identity) in the open reading frame encoded by exons 4 through 7 in the 3’ region of the gene but differ in the 5’ untranslated region encoded by exons 1 through 4 [11–12]. These three isoforms can differ in tissue expression, function, and biochemical properties [12]. FRα is the most widely studied FR protein isoform and mediates the transfer of one-carbon units by folate, which is necessary for proper synthesis of purines, pyrimidines and therefore the synthesis of DNA and RNA. Furthermore, folate is also involved in the methylation of DNA, proteins and phospholipids [13]. Related to its crucial metabolic roles, FRα overexpression or deficiency, through folate uptake, can result in a faster or slower cell growth rate and lead to abnormally methylated genes and faulty DNA replication [13, 14].

FRα is expressed at elevated levels in normal pneumocytes, thymocytes and renal tubules. However, it is dysregulated in a wide variety of human malignancies [15], such as pituitary [16], lung [17–20], breast [21], colorectal [22, 23], and ovarian cancers [24–26]. Furthermore, FRα expression levels have been associated with prognosis in these types of cancers. To date, however, the association of FRα expression with clinicopathological characteristics and prognosis in PDAC has not been clearly defined. In this study, we analyzed FRα expression levels in resected PDAC specimens and PDAC cell lines in order to define the potential significance of FRα expression in PDAC tumors relative to the clinicopathological characteristics and prognosis of this disease.

## RESULTS

### Clinicopathologic features of the overall patient cohort

Samples from 156 patients who underwent pancreatic resection at our institution were analyzed. However, of those, samples from 16 patients were excluded from further analysis: 9 for insufficient number of cores, and 7 for inadequate follow-up. The clinical characteristics of the patients are summarized in Table 1. The median age at the time of pancreatectomy was 70.0 years (interquartile range: 60-76), and 77 (55.0%) patients were female. The majority of patients had stage IIB disease (69.3%) and 85 (60.7%) had moderately differentiated tumors. Post-operatively, 65 patients (46.4%) received adjuvant chemo-radiotherapy, 20 (14.3%) received chemotherapy alone and one (0.7%) radiotherapy alone.

**Table 1:** Correlation of PDAC FRα expression intensity and clinicopathological characteristics in 140 PDAC patients

	FRα expression intensity								p-value
	Low		Intermediate		High		Total		
	N=22 (16%)		N=73 (52%)		N=45 (32%)		N=140		
	N	%	N	%	N	%	N	%	
Gender									
Male	12	54.5%	31	42.5%	20	44.4%	63	45.0%	0.605
Female	10	45.5%	42	57.5%	25	55.6%	77	55.0%	
Age, y (median, IQR)	73	63-76	66	60-74	70	61-76	70	60-76	
Race									
White	22	100.0%	68	93.2%	42	93.3%	132	94.3%	0.613
Black	0	0.0%	0	0.0%	0	0.0%	0	0.0%	
Asian	0	0.0%	1	1.4%	1	2.2%	2	1.4%	
Hispanic	0	0.0%	4	5.5%	1	2.2%	5	3.6%	
Other	0	0.0%	0	0.0%	1	2.2%	1	0.7%	
CACI (median, IQR)	4	3-5	3	2-4	3	3-4	3	2-4	
BMI, kg/m^2^ (median, IQR)	24.4	23.5-27.9	25.3	23.2-28.5	26.5	22.1-30.1	25.3	23.2-28.8	
Smoking (Yes, ever)^a^	20	90.9%	45	61.6%	10	22.2%	75	53.6%	<0.001
Alcohol (Yes, ever)^a^	16	72.7%	23	31.5%	12	26.7%	51	36.4%	0.001
LOS, d (median, IQR)	7	6-9	7	6-11	7	6-9	7	6-10	
Size, cm (median, IQR)	3.3	2.5-4.8	3.4	2.5-4.3	3.3	2.5-4.0	3.3	2.5-4.3	
Grade									
Well differentiated	1	4.5%	2	2.7%	1	2.2%	4	2.9%	0.388
Moderately differentiated	10	45.5%	50	68.5%	25	55.6%	85	60.7%	
Poorly differentiated	11	50.0%	19	26.0%	17	37.8%	47	33.6%	
Undifferentiated	0	0.0%	2	2.7%	2	4.4%	4	2.9%	
Lymphnodes positive	18	81.8%	48	65.8%	29	64.4%	95	67.9%	0.308
TNM Stage									
IA	0	0.0%	4	5.5%	0	0.0%	4	2.9%	0.216
IB	1	4.5%	9	12.3%	4	8.9%	14	10.0%	
IIA	3	13.6%	10	13.7%	12	26.7%	25	17.9%	
IIB	18	81.8%	50	68.5%	29	64.4%	97	69.3%	
Lymphovascular invasion^a^	18	81.8%	43	58.9%	17	37.8%	78	55.7%	0.002
Perineural invasion	20	90.9%	62	84.9%	43	95.6%	125	89.3%	0.187
Resection type									
R0	15	68.2%	50	68.5%	24	53.3%	89	63.6%	0.223
R1	7	31.8%	23	31.5%	21	46.7%	51	36.4%	

^a^Statistically significant differences between groups. CACI: age-adjusted Charlson Comorbidity Index; IQR: interquartile range.

### FRα expression in PDAC specimens

**Figure 1:**
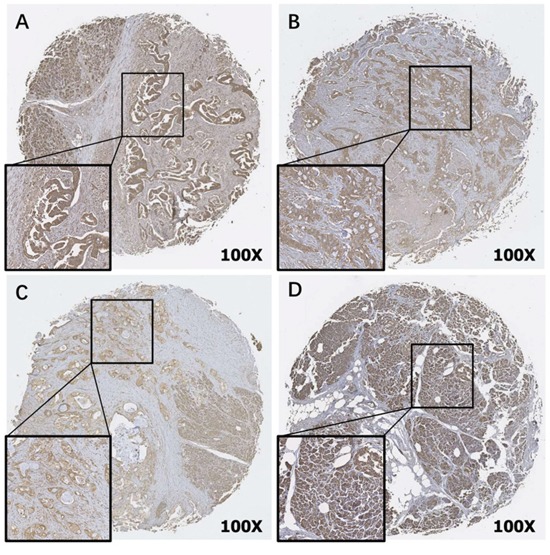
Immunohistochemical detection of Folate Receptor alpha (FRα) in TMAs of PDAC **(A-C)** and paraneoplasic normal pancreatic tissue **(D)** (original magnification 100X). Representative staining patterns of High **(A)**, Intermediate **(B)** and Low **(C)** FRα expression intensity are shown. Paraneoplasic normal pancreatic tissue **(D)** was stained as a positive control..

The level of expression of FRα in tumor specimens obtained from the 140 PDAC patients was determined by IHC analysis as previously detailed. FRα expression intensity was found to be low in 22 (16%), intermediate in 73 (52%) and high in 45 (32%) specimens from the 140 PDAC patients (Figure 1A-C). The vast majority of samples (123, 88%) showed both membranous and cytoplasmic staining, whereas in the remaining cases (17, 12%) only cytoplasmic staining was detected. Compared with paraneoplasic tissues, membranous and/or cytoplasmic expression intensity of FRα was markedly lower in PDAC (Figure 1B-1C vs 1D). The inter-rater reliability for the FRα expression intensity assessment was almost perfect (Cohen's κ =0.81, p<0.001).

### The relationship between the intensity of FRα expression in PDAC patient specimens and the patients’ clinicopathology characteristics

Smoking and alcohol consumption were significantly associated with lower FRα expression intensity. The percentages of smokers in each FRα expression group were as follows: low FRα expression intensity: 20 out of 22 (90.9%), intermediate: 45 out of 73 (61.6%), high: 10 out of 45 (22.2%) (p<0.001). The percentages of alcohol users in each FRα expression group were as follows: low FRα expression intensity: 16 out of 22 (72.7%), intermediate: 23 out of 73 (31.5%), high: 12 out of 45 (26.7%) (p<0.001) (Table 1). Additionally, lymphovascular invasion was significantly associated with lower FRα expression intensity (low: 81.8%, intermediate: 58.9%, high: 37.8%, p=0.002) (Table 1). There was no significant difference between the three FRα expression level groups in terms of gender, age, race, CACI, BMI, LOS, tumor size, grade, lymph node status, TNM stage, PNI or resection margin status of the PDAC patients.

### FRα expression intensity as a prognostic factor of overall survival

**Figure 2:**
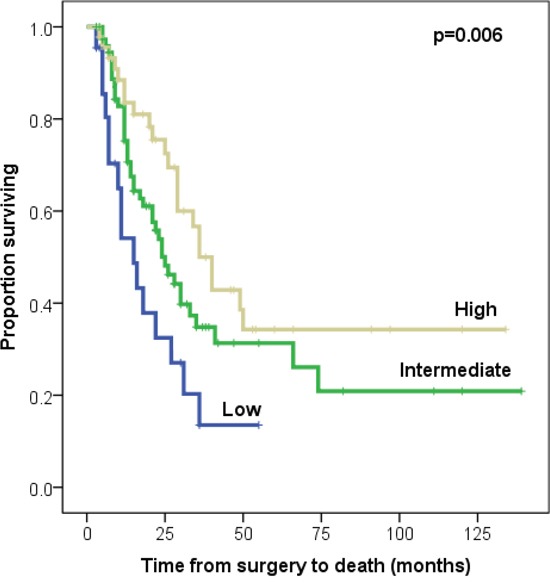
Kaplan-Meier survival curves of low vs. intermediate vs. high FRα expression intensity in 140 PDAC patients

Median overall survival (OS) and median follow-up for the complete cohort were 27.6 months (range 2.6-138.8) and 20.4 months (range 2.6-138.8), respectively. Median OS of patients in the low, intermediate and high FRα expression intensity groups was 15.1 months (range 2.6-59.4), 24.0 months (range 2.9-138.8) and 36.3 months (range 3.4-133.2), respectively. One-, 3-, and 5-year OS rates were 56%, 21%, 13% for the low, 81%, 34%, 31% for the intermediate and 86%, 53%, and 33% for the high FRα intensity groups, respectively (Table 2). In univariate analysis, high FRα intensity was associated with better OS (p=0.006) (Figure 2). In multivariate analysis, FRα expression intensity (HR=0.61; p=0.026) and CACI score (HR=1.16; p=0.010) were shown to be independent predictors of OS for the entire cohort. Adjuvant chemo-radiotherapy, chemotherapy alone, or radiotherapy alone were not associated with survival in either univariate or multivariate analyses.

**Table 2:** Distribution of FRα expression with the overall survival rate in PDAC

FRα expression level	Median survival (months)	95% CIs	IQR	Range	1y OS (%)	3y OS (%)	5y OS (%)
Low	15.1	7.2-23.0	7.1-31.1	2.6-54.9	56	21	13
Intermediate	24.0	17.5-30.6	13.0-73.9	2.9-138.8	81	34	31
High	36.3	29.4-43.2	24.6-n/a	3.4-133.2	86	53	33
Total	27.6	22.4-32.8	12.2-73.9	2.6-138.8	79	38	28

CI: confidence interval; IQR: interquartile range

### Expression of FRα on the surface of patient-derived PDAC cell lines and long-established PDAC cell lines

**Figure 3:**
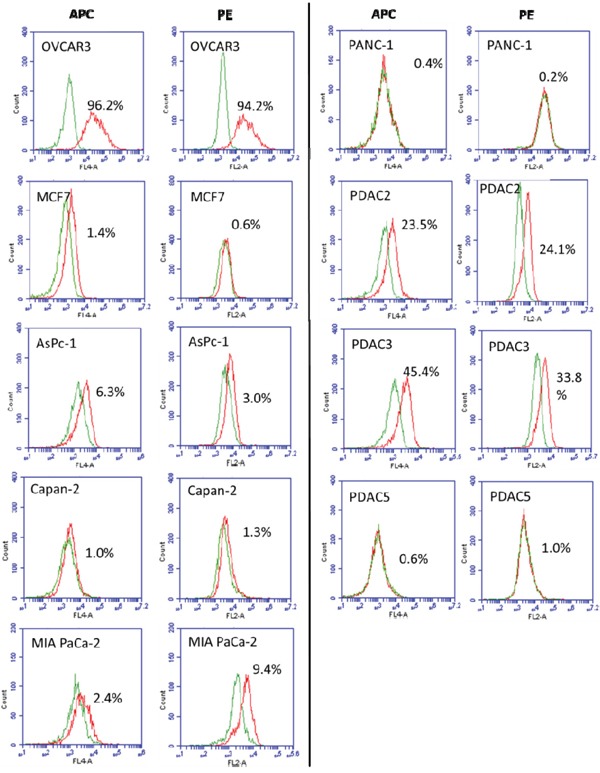
Analysis of FRα expression on human PDAC cell lines AsPC-1, Capan-2, MIA PaCa-2, PANC-1 PDAC2, PDAC3, and PDAC5 Cells were stained with either APC- or PE- conjugated FRα-specific antibodies. Stained cells were subjected to flow cytometry analysis on a BD ACCURI C6 flow cytometer using BD CSampler software (Becton Dickinson and Company, San Jose, CA). OVCAR3 and MCF7 cells were used as positive and negative control, respectively. The percentage of cells stained with the FRα-specific antibodies is shown in each histogram (Green: conjugated negative antibody control; Red: conjugated FRα-specific antibodies).

To facilitate future *in vitro*-based studies in development of antibody or Chimeric Antigen Receptor (CAR) T-Cell based FRα –targeted immunotherapies, we determined if FRα is expressed on cell surface of PDAC cell lines. Seven human PDAC cell lines, AsPC-1, Capan-2, MIA PaCa-2, PANC-1 PDAC2, PDAC3, and PDAC5, were analyzed for cell surface expression of FRα by flow cytometry analysis using APC- and PE-conjugated FRα-specific antibodies, recognizing the same epitope. When the APC-conjugated antibody was used, FRα positive cells were present at a frequency of 6.3%, 1.0%, 2.4%, 0.4%, 23.5%, 45.4%, and 0.6%, in AsPC-1, Capan-2, MIA PaCa-2, PANC-1 PDAC2, PDAC3, and PDAC5 cell lines, respectively. When the PE-conjugated antibody was employed, similar results were obtained for FRα positive cells on the above PDAC cell lines as 3.0%, 1.3%, 9.4%, 0.2%, 24.1%, 33.8%, and 1.0%, respectively (Figure 3).

## DISCUSSION

In this study, which to our knowledge is the first of its kind to evaluate the prognostic value of FRα expression in PDAC, high FRα expression intensity in surgically removed PDAC specimens was found to be significantly associated with favorable prognosis. Previous reports have also shown correlations of FRα expression with prognosis in other malignancies. Interestingly, high FRα expression in lung adenocarcinoma was found to be associated with early stage disease and favorable prognosis [20, 27]; in contrast, high FRα expression was shown to be associated with poor prognosis in breast cancer [28, 29] (but not relative to breast cancer brain metastases) [21], and with poor disease-free and overall survival, as well as chemoresistance in ovarian cancer [30–32]. In the present study, although we found that FRα expression level was an independent predictor of survival, we did not find a correlation with tumor stage or grade. This result may reflect the small sample size of patients with stage 1a disease (only 4 cases), and with differentiated and undifferentiated tumors (4 cases each).

The reasons for the discrepancies between tumor types, as well as the mechanisms by which FRα is implicated in cancer progression remain largely unknown. It has been hypothesized that FRα plays a role in malignancies through both folate-related and unrelated mechanisms. While folate enters the cells in normal tissues mainly through the reduced folate receptor (RFC), in rapidly growing cells, such as malignant cells, the upregulation of FRα accelerates folate uptake, thus facilitating growth [27]. Additionally, FRα can translocate to the nucleus and act as a transcription factor for developmental genes [33], or activate signaling pathways by inducing STAT3 activation [34, 35] and LYN tyrosine kinase phosphorylation [24, 36]. However, FRα expression might simply be a “bystander” with no effect on cancer progression [15]. Indeed, in tumors in which high FRα expression confers better prognosis – lung adenocarcinoma and PDAC – FRα expression might just mirror the normal FRα expression of the non-cancerous cells of origin [27].

All the PDAC specimens analyzed in this study expressed FRα, with more than 80% of cases presenting intermediate or high expression levels. As a result, FRα might be a promising target for novel therapeutic or diagnostic strategies. Several FRα-based diagnostic and treatment modalities have been recently described in a variety of other malignancies and could potentially be applied in PDAC. FRα-specific imaging probes, small molecules targeting FRα, drug conjugates [37], CAR T cells, vaccines, monoclonal antibodies and bispecific antibodies could all be potentially used for the improved diagnosis and treatment of PDAC [24]. Tempering these approaches, however, is the finding that normal and paraneoplastic pancreatic tissues were highly positive for FRα expression, in accordance with previous reports in which 40%-100% of normal pancreatic tissues were FRα positive [27, 38]. Although this observation raises concerns about the specificity of FRα-targeting regimens in PDAC, no pancreas-specific adverse effects have been reported in a number of clinical trials evaluating the safety and efficacy of such drugs [24].

The results of this study also established that low FRα expression in PDAC tissues correlated with smoking and alcohol consumption. Other studies have strongly suggested that smoking is an important factor which promotes pancreatic cancer. Together these findings suggest that FRα expression may be suppressed by smoking and alcohol. Determining the mechanisms underlying the effect of smoking compounds and/or alcohol on FRα expression may provide important insights into the pathogenesis and progression of pancreatic cancer. Of note, Iwakiri et al also demonstrated a downregulation of FRα in heavy smokers [20]. Given that diabetes and meat-rich diet have also been associated with poor prognosis in PDAC, future studies should attempt to elucidate the correlation between these factors and FRα expression levels.

In order to facilitate the conduction of experiments evaluating novel anti-cancer drugs for PDAC, we analyzed by flow cytometry the expression of FRα on both long established (commercially available) and recently established patient-derived PDAC cell lines. Whereas, the long established cell lines AsPc-1, Capan-2 and PANC-1 were negative for FRα expression, the recently established cell lines PDAC2 and PDAC3 were positive. Of note, mRNA expression of the FOLR1 gene has also been found to be low for AsPc-1, MIA PaCa-2 and PANC-1 cell lines [39]. Our results provided fundamental information on FRα cell surface expression range at protein level on a panel of PDAC cell lines. This might serve well as a guide for the choice of PDAC cell lines to be used for mechanistic study of FRα expression in PDAC cells and FRα targeted approaches for PDAC.

Although the results of this study are limited due to the use of a polyclonal antibody for IHC, and from its single center design, they demonstrate that higher expression levels of FRα are predictive of a favorable prognosis in PDAC and FRα may represent a promising target for novel therapeutic strategies, including immunotherapy for PDAC.

## MATERIALS AND METHODS

### Tissues and tissue microarrays (TMAs)

The normal and tumor specimens from primary PDAC lesions were obtained from patients who underwent either pancreaticoduodenectomy or distal pancreatectomy at the Massachusetts General Hospital, between the years 1998 to 2012. Clinicopathologic information available included patient age, gender, race, age-adjusted Charlson Comorbidity Index (CACI) [40], Body Mass Index (BMI), smoking, alcohol consumption, length of hospital stay (LOS), tumor size, tumor grade, tumor stage, lymphovascular invasion, perineural invasion, resection margin status (R0 or R1, defined as margins>1mm or </=1mm, respectively) [41] and survival data. Surgical specimens were processed within 25 minutes after surgical excision. Tissue samples were fixed in 20% buffered formalin and embedded in paraffin following conventional procedures. PDAC tumors were confirmed histopathologically by a gastrointestinal pathologist (V. Deshpande) according to the AJCC (7th Edition) and the WHO classification systems. A TMA was constructed with three to five 3-mm cores per patient before the procedure of immunohistochemistry. This study was approved by the Institutional Review Board (protocol number: 2002P000154).

### Cell lines

The PDAC cell lines AsPC-1, Capan-2, MIA PaCa-2 and PANC-1 were purchased from ATCC; PDAC2, PDAC3 and PDAC5 were generated from metastatic ascites fluid from patients receiving treatment at the Department of Surgery, Massachusetts General Hospital [42]. The cell lines OVCAR3 and MCF7 were purchased from ATCC and used as a positive [43] and negative [44] control for FRα expression, respectively.

### Immunohistochemical (IHC) staining

TMA blocks of tumor specimens were cut into 5-μm sections and were used as substrates in IHC reactions (Normal pancreas tissue and stroma were used as the positive and negative control, respectively). IHC staining was performed with Rabbit Polyclonal (IgG) to Human FOLR1/Folate Receptor Alpha Antibody (LS-B5727, LSBio) using the EnVision+ system (Dako) Kit. Briefly, TMA specimens were sectioned at 5μm onto positively-charged glass slides and heated for 12 hours at 65°C. Slides were deparaffinized in 3 sequential baths of xylene for 5min each, transferred to 2 sequential baths of 100% alcohol for 30sec each, followed by one bath of 95% alcohol for 30sec, followed by 2 sequential baths of 75% alcohol for 30sec each and then rinsed for 5min in deionized (DI) water. For antigen retrieval, slides were incubated for 20min in the diluted Target Retrieval Solution 10X Concentrate (DAKO, S1699) in which the container incubation reaches a maximum of 100°C and then cooled for 1 hour and 30 min down to room temperature (RT). After cooling to RT, slides were placed into 3% Hydrogen Peroxide Solution (Sigma) for 20min at RT and subsequently washed in 3 sequential baths of Tris Buffered Saline/0.1% Tween-20 wash buffer (TBST) for 5min each. After washing in TBST, slides were incubated with 1% BSA/5% NHS in TBST (Blocking Reagent) for 1 hour at RT. Subsequently, slides were incubated overnight at 4°C with Rabbit Polyclonal (IgG) to Human FOLR1 antibody (LS-B5727, LSBio) at a concentration of 5μg/mL diluted in blocking reagent. After washing in TBST for 5 sequential baths, slides were incubated with DAKO envision + system -HRP labeled polymer anti-rabbit at RT for 45 min. Following 3 sequential baths, slides were incubated with Dako Liquid DAB+ Substrate Chromogen System for 10sec and counterstained with hematoxylin (Dako) for 30sec, all incubations being performed at RT. After dehydration, slides were covered with cover glasses.

### IHC scoring method

Staining intensity of stained tumor cells in each lesion was reviewed independently by two investigators (LC and TM) and was confirmed by a gastrointestinal pathologist (Qi Shen) using a Nikon Eclipse 80i microscope (Nikon, Japan). Investigators were blinded to the patients’ characteristics and clinical outcomes. The locations of FRα staining were marked as: cytoplasm, membrane, cytoplasm and membrane, using 10x, 20x and 40x objectives. The staining was scored based on the intensity as 1-Low (negative or weak staining); 2-Intermediate (moderate staining); and 3-High (strong staining). Normal pancreatic cells, which were stained with high intensity in all specimens, were used as a control of high intensity. Tumor stroma, where staining was absent in all specimens, was used as a negative control. Staining intensity in-between that of normal pancreatic cells and stroma was considered “intermediate”. Since all tumor cells in the same lesion had the same staining intensity, the percentage of stained cells for each intensity category was not taken into account. The mean intensity was calculated as (sum of intensity scores / numbers of cores analyzed) for each patient. Thereafter, the mean was rounded to the closest integer and the respective score was given (1-low, 2-intermediate, 3-high). Patients for whom less than three cores were available after the staining process were excluded from further analysis.

### Flow cytometry analysis

Cells were stained with antibodies specific to FRα by flow cytometry as previously described [45]. Two mouse IgG monoclonal anti-FRα antibodies recognizing FRα Arg25-Met233 were independently used. One was conjugated with PE (FAB5646P, R&D Systems) and the other with APC (LS-C129132, LSBio). We used both antibodies to test our flow cytometry assay reproducibility. Briefly, cells (1 × 10^5^) were resuspended in phosphate-buffered saline (PBS; pH 7.4) (Gibco) containing 2% BSA in 5ml Polystyrene Round-Bottom tubes (BD Falcon, BD-352001) and incubated with the anti- FRα antibodies. After washing twice with PBS, cells were resuspended in PBS containing 2% PFA for flow cytometry analysis. Five thousand gated events were analyzed by flow cytometry on a BD ACCURI C6 using BD CSampler software (Becton Dickinson and Company, San Jose, CA). The ovarian cancer cell line OVCAR3 and the breast cancer cell line MCF7 were used as the positive and negative control, respectively, for FRα expression.

### Statistical analysis

The chi-square test was used to compare categorical variables between groups, while one-way ANOVA was used for continuous variables. The inter-rater reliability was assessed using Cohen's κ. Overall survival (OS) was calculated as time between the date of operation and the date of death (event) or last follow up (censored). Survival curves were plotted with the Kaplan-Meier method and differences in survival between groups was analyzed utilizing the log-rank test. Multivariate survival analyses were conducted using a stepwise backward Cox proportional hazards survival model. A p-value less than 0.05 was considered to be statistically significant and all tests used were 2-tailed. All statistical analyses were performed with IBM SPSS Statistics for Windows, Version 21.0 (IBM Corp., Armonk, NY).

## Abbreviations

AJCC: American Joint Committee on Cancer
ATCC: American Type Culture Collection
BMI: Body mass index
BSA: Bovine serum albumin
CACI: age-adjusted Charlson Comorbidity Index
CAR T-Cells: Chimeric Antigen Receptor T-Cells
CI: confidence interval
DNA: Deoxyribonucleic acid
FBP: Folate binding protein
FRα: Folate receptor alpha
FRβ: Folate receptor beta
FRγ: Folate receptor gamma
IHC: Immunohistochemistry
IQR: interquartile range
LOS: Length of hospital stay
PBS: Phosphate buffered saline
PDAC: Pancreatic ductal adenocarcinoma
PNI: Perineural invasion
RFC: Reduced folate receptor
RNA: Ribonucleic acid
RT: Room temperature
OS: Overall survival
TBS: Tris-buffered saline
TMA: Tissue microarray
